# Population Pharmacokinetics of Risperidone and Paliperidone in Schizophrenia: A Systematic Review

**DOI:** 10.3390/ph18050698

**Published:** 2025-05-08

**Authors:** Ana Carrascosa-Arteaga, Ricardo Nalda-Molina, Patricio Más-Serrano, Amelia Ramon-Lopez

**Affiliations:** 1 School of Pharmacy, Miguel Hernández University, 03550 San Juan de Alicante, Spain; a.carrascosa@umh.es (A.C.-A.); mas_pat@gva.es (P.M.-S.); aramon@umh.es (A.R.-L.); 2 Alicante Institute for Health and Biomedical Research (ISABIAL-FISABIO Foundation), 03010 Alicante, Spain; 3 Clinical Pharmacokinetics Unit, Pharmacy Department, Alicante University General Hospital, 03010 Alicante, Spain

**Keywords:** schizophrenia, antipsychotic agents, risperidone, paliperidone palmitate, drug monitoring, pharmacokinetics

## Abstract

**Background:** The primary treatment of schizophrenia is pharmacotherapy with antipsychotic agents, such as risperidone and paliperidone. Population pharmacokinetic (PopPK) modelling plays a crucial role in optimising therapy by predicting of plasma concentrations, therapeutic efficacy, and the risk of adverse effects using model informed precision dosing. **Objectives:** This systematic review examined the PopPK models of risperidone and paliperidone in patients diagnosed with schizophrenia based on the available scientific evidence. **Methods:** A systematic review of the health science databases was conducted. The inclusion criteria were original articles published in peer-reviewed journals, studies focusing on the development of original PopPK models of risperidone and paliperidone, and clinical studies. The exclusion criteria were full-text articles that could not be retrieved; studies not including subjects diagnosed with schizophrenia or schizoaffective disorders; and studies that did not investigate risperidone or paliperidone. **Results:** A total of 19 studies developing PopPK models were analysed, including one- or two-compartment PopPK model structures. Interindividual variability in the pharmacokinetic parameters was shown to be influenced by factors such as CYP2D6 activity, renal function, body mass index, and sex. Parameter estimation revealed high variability in clearance and volume of distribution. **Conclusion:** Numerous PopPK models for risperidone and paliperidone have been published with a detailed characterisation of absorption, metabolism, and elimination. Therefore, future research should focus on the external validation of these models to facilitate their integration into clinical practice and optimise individualised dosing, ultimately improving treatment efficacy and safety across diverse patient populations.

## 1. Introduction

Schizophrenia is a chronic mental illness whose worldwide prevalence is 1% [1,2,3,4,5]. It is characterised by a wide variety of symptoms classified as positive and negative that entail a significant degree of disability in the patient and have a negative impact on occupational productivity, social relationships, and self-care [4,5,6,7]. These factors lead to a higher rate of unemployment, isolation, mortality, and deterioration of the patient’s quality of life [1,2,4,7].

Although this pathology must be addressed from a multidisciplinary perspective, pharmacotherapy remains the primary approach, with monotherapy using antipsychotics representing the first-line treatment [2,4,7,8]. The choice of antipsychotic is made based on the characteristics of both the drug and the patient, taking into account the patient’s preferences, dosing schedule, route of administration, the side effect profile, pharmacokinetics (PK), and cost of treatment [1,4,5]. Currently, second-generation antipsychotics are the most commonly used treatment, as they are associated with fewer extrapyramidal effects than first-generation antipsychotics but with greater metabolic effects [1,9,10].

Risperidone and paliperidone are available in oral formulations and long-acting injectable (LAI) formulations; additionally, risperidone is available as a solution and in orodispersible tablet formulations [5,11,12,13,14,15,16,17,18]. LAI formulations of risperidone and paliperidone utilise microsphere-based delivery systems with distinct release mechanisms. LAI paliperidone is formulated as microspheres containing paliperidone palmitate, a prodrug obtained through the esterification of paliperidone. Following intramuscular injection, the paliperidone palmitate undergoes enzymatic hydrolysis by esterases within the muscle tissue, releasing free paliperidone. In this system, particle size plays a key role in determining the sustained-release properties. On the other hand, LAI risperidone is formulated as microspheres of risperidone encapsulated within a biodegradable polymer, which progressively degrades after administration, enabling controlled drug release [19,20,21,22].

One of the primary objectives of these LAI formulations is to improve patient adherence. The increased adherence observed with LAI use leads to a reduction in hospitalisation duration, a decrease in the number of relapses, and a reduction in healthcare resource utilisation [2,8,10,23,24,25]. In addition, LAI formulations reduce dosing frequency, enable a more objective assessment of adherence, increase bioavailability (F) by bypassing first-pass hepatic metabolism, and provide more stable plasma concentrations [10,23,24,26].

Plasma concentrations of active moiety (risperidone and paliperidone plasma concentrations) determine both the therapeutic efficacy and toxicity of the drug, as a correlation between plasma concentration and dopamine D2 receptor occupancy has been demonstrated [1,5,27,28,29]. Regarding risperidone, it is important to note that some authors attribute its pharmacological activity to its active fraction [30,31,32,33]. Clinical efficacy has been observed when dopamine D2 receptor occupancy exceeds 65%, while an occupancy above 80% increases the likelihood of experiencing adverse reactions and extrapyramidal symptoms [1,5,27,34,35]. This occupancy percentage corresponds to plasma concentration levels between 20 and 60 ng/mL for both risperidone and paliperidone, which, according to some authors, could be considered a PK target to achieve an optimal clinical response [27,29,36].

Population pharmacokinetic (PopPK) models are mathematical representations of the absorption, distribution, metabolism, and elimination in a patient population. PopPK models aim to characterise variability in drug concentrations within a patient group. These PopPK models are often compartmental models, which conceptualise the body as one or more interconnected compartments. Model-informed precision dosing (MIPD) is an advanced strategy for optimising individualised pharmacotherapy. It is based on PopPK and pharmacodynamic (PD) mathematical and statistical models, integrating patient-specific clinical and demographic data. Using Bayesian estimation, MIPD enables the prediction of personalised dosing strategies by accounting for inter- and intra-individual variability in pharmacological parameters. Its application in drug development and clinical practice has proven to enhance treatment safety and efficacy, establishing itself as a key tool in precision medicine.

The objective of this systematic review is to perform an analysis of the original PopPK models of risperidone and paliperidone published in the scientific literature and to provide a critical evaluation of the current scientific knowledge.

## 2. Methods

### 2.1. Design

This was a descriptive study and critical analysis of studies retrieved through a systematic review. The structure of this review followed the Preferred Reporting Items for Systematic Reviews and Meta-Analysis (PRISMA) guidelines [37] (Supplementary Table S1) and the methodological framework proposed for scoping studies [38,39].

### 2.2. Source of Data Collection

The data were obtained through direct consultation and access, via the Internet, to the following bibliographic databases in the field of health sciences: MEDLINE (via PubMed), Embase, Cochrane Library, Scopus, PsycINFO, Web of Science, and Latin American and Caribbean Health Sciences Literature (LILACS). The published articles were analysed and retrieved from these bibliographic databases.

### 2.3. Information Processing

Search terms were selected using the Thesaurus of Health Sciences Descriptors (DeCS) developed by the Latin American and Caribbean Center on Health Sciences Information (BIREME) and equivalent terms established by the US National Library of Medicine, Medical Subject Headings (MeSH). The MeSH descriptors “Schizophrenia”, “Risperidone”, “Paliperidone Palmitate”, “Drug Monitoring”, and “Pharmacokinetics” were considered suitable. Likewise, these terms were used to query the database using the title and abstract field (Title/Abstract). It was not necessary to use filters (limits). The registration number of the protocol in PROSPERO is CRD420251042068.

From the study of both Thesaurus and their indexing records (Entry Terms), the following search equations were considered appropriate:

POPULATION: Schizophrenic Subjects

(Schizophrenia[MeSH Terms] OR Schizophrenia*[Title/Abstract] OR “Dementia Praecox”[Title/Abstract] OR “schizophrenic disorder*”[Title/Abstract])

INTERVENTION: Risperidone or Paliperidone Palmitate Therapy

(Risperidone[MeSH Terms] OR Risperidone[Title/Abstract] OR “Risperdal Consta”[Title/Abstract] OR Risperidal[Title/Abstract] OR “R 64766”[Title/Abstract] OR R64766[Title/Abstract] OR “R-64766”[Title/Abstract] OR “Paliperidone Palmitate”[MeSH Terms] OR Paliperidone[Title/Abstract] OR “9-OH-risperidone”[Title/Abstract] OR “9 OH risperidone”[Title/Abstract] OR “3-(2-(4-(6-fluoro-3-(1,2-benzisoxazolyl))-1-piperidinyl)ethyl)-6,7,8,9-tetrahydro-9-hydroxy-2-methyl-4H-pyrido(1,2-a)pyrimidin-4-one”[Title/Abstract] OR “9-Hydroxy-risperidone”[Title/Abstract] OR “9 Hydroxy risperidone”[Title/Abstract] OR “9-Hydroxyrisperidone”[Title/Abstract] OR “9 Hydroxyrisperidone”[Title/Abstract] OR Invega[Title/Abstract] OR “Invega Sustenna”[Title/Abstract] OR “R 76477”[Title/Abstract] OR “R-76477”[Title/Abstract] OR R76477[Title/Abstract])

OUTCOME: Original Population Pharmacokinetic Models

(Drug Monitoring[MeSH Terms] OR “Drug Monitoring”[Title/Abstract] OR “Therapeutic Drug Monitoring”[Title/Abstract] OR Pharmacokinetics[MeSH Terms] OR pharmacokinetic*[Title/Abstract] OR “drug kinetic*”[Title/Abstract])

This strategy was subsequently adapted to the specific characteristics of each of the other databases consulted, with the search being carried out from the first date available in each of the selected databases until February 21, 2025 (available in Supplementary Table S2). Additionally, a complementary search strategy was performed to reduce the possibility of publication bias by manually searching the reference lists of the articles selected for the review. Likewise, experts in the subject under study were contacted to determine the possible existence of grey literature (materials and research produced by organisations outside traditional commercial or academic publications disseminated through other distribution channels).

### 2.4. Final Selection of Articles

For the review and critical analysis, articles meeting the following criteria were selected:

Inclusion: original articles published in peer-reviewed journals; articles focusing on the development of original compartmental PopPK models of risperidone and paliperidone; and clinical studies, irrespective of the study design.

Exclusion: articles for which the full text was unavailable; articles not written in English or Spanish; articles that did not include subjects with schizophrenia or schizoaffective disorders; and articles that did not include risperidone or paliperidone in their studies.

There were no restrictions on publication date or publication status. The selection of relevant articles was performed by two authors of the present review (A.C.-A. and A.R.-L.). To validate the inclusion of the articles, inter-rater agreement was assessed using the kappa index, which had to be greater than 0.60 [40,41]. In case of discrepancies, a third reviewer (R.N.-M.) was responsible for reaching a resolution and subsequent consensus among all the authors.

### 2.5. Data Extraction

Data from eligible articles were collected to systematise and facilitate the interpretation of the results, which were presented and summarised in tables. Data were extracted by one reviewer and verified by a second reviewer. The following information was extracted: general information of study (first author, country of study, type of study, year of publication, and duration of study); population characteristics (age, body mass index (BMI), weight, sex, height, race or ethnicity, creatinine clearance (CL_CR_), comedication, CYP2D6 metaboliser type, and diagnosis); study design (number of patients and collected concentrations used for model building, dosage regimens, drug and type of formulation, injection site, number and arms of the clinical trials, bioanalytical method used to analyse drug concentration, and lower limit of quantification (LLOQ)). Dose regimens are presented as median and/or its range, and results (software used for PopPK analysis, model structure, estimation method, model evaluation, PK parameter estimates and covariates relationships, inter-individual variability (IIV), inter-occasion variability (IOV), and residual unexplained variability). The IIV was presented as the coefficient of variation (CV%). The values of the variables age, BMI, weight, and CL_CR_ collected from the different studies were measured using various methods. Therefore, it was decided to transform the values reported as median and range into approximations of the median and standard deviation using statistical methods. This transformation aimed to homogenise the results and facilitate their comparison.

## 3. Results

The systematic review identified a total of 3147 publications: 446, 243, 1037, 296, 142, 978, and 5 from MEDLINE (via PubMed), Cochrane Library, Scopus, Embase, psycINFO, Web of Science, and LILACS, respectively. After removing duplicates, applying the inclusion and exclusion criteria, and consulting the bibliographic lists of the selected articles from the search strategy, 19 original articles were included in this review. The inter-rater agreement for the selected studies was 0.895 (*p* < 0.001), according to the kappa index.

**Figure 1:**
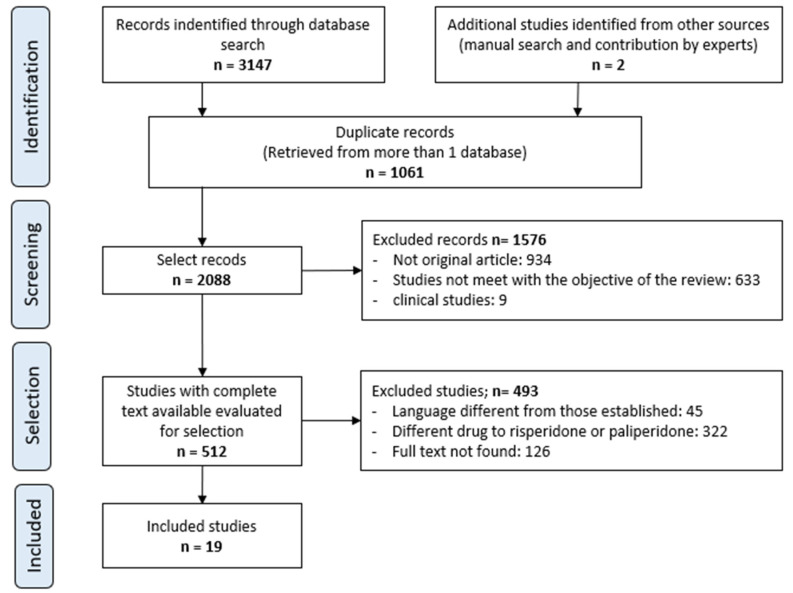
Selection procedure of the studies.

The process of study selection is presented in a flowchart in Figure 1.

All included studies developed a new PopPK model in schizophrenia patients treated with risperidone or paliperidone. The summary of the characteristics of each study is presented in Table 1, whereas the model PopPK parameters are summarised in Table 2.

### 3.1. Study Design

Regarding the study design, 14 of the 19 studies were multicentre [41,42,43,44,45,46,47,48,49,50,51,52,53,54,55,56,58,59,60], and all studies performed a retrospective analyses of previously conducted clinical trials, except for one [55], which relied on data from three studies. Two of these were longitudinal studies, while the other was a retrospective study. Only six studies were based on data from a single clinical trial [42,47,52,54,56,57], whereas the others utilised data from multiple trials.

The duration of the included studies varied considerably, ranging from three weeks to two years; however, in several articles, this information was either unavailable or incomplete for all the studies included in the article.

### 3.2. Population of the Studies

Of the retrieved PK studies, six studies were conducted in different countries [41,42,43,45,46,49], and the remaining studies were conducted in the United States and Europe. Eight studies exclusively examined patients with a diagnosis of schizophrenia [42,46,47,48,49,51,52,57], while the remaining studies incorporated patients with a range of psychiatric disorders, including bipolar disorder, conduct disorder, and Alzheimer’s disease [41,43,44,45,50,53,54,55,56,58,59]. The majority of the reviewed studies restricted enrolment only to subjects in a clinically stable condition [42,47,49,51,53,54,57]. Only two studies considered both stable and acutely patients [45,48], while one study focused exclusively on acutely patients [52]. The remaining studies did not disclose the clinical status or severity of the patients included. In addition, four of them included healthy subjects [41,43,44,45], and one study included other pharmacological agents in addition to risperidone and paliperidone, such as haloperidol, ziprasidone, or olanzapine [48].

The mean age of the patients included in the studies was similar across studies. A total of 17 studies were conducted exclusively in adult patients, while only 2 included paediatric and adolescent populations [43,44]. The mean weight and BMI values of the patients included in the studies ranged from 74.3 to 89.7 kg and 25.9 to 34.3 kg/m^2^, respectively. Regarding CL_CR_, the lowest mean value was recorded in the study by Vandenbergue et al. [55], reporting a value of 115 mL/min. Several studies did not report these values. The sample size in each study ranged from 45 to 1471 patients, while the number of observations for model building ranged from 178 to 15,754. The number of observations used for model development was not reported in nine studies [41,44,46,48,54,57] of the PopPK models. Similarly, the number of patients included in the analysis was unavailable in three studies [41,44,46]. In addition, no data were available regarding the characteristics of the population used for the development of seven PopPK models [41,44,46,48].

### 3.3. PopPK Models

Regarding the type of formulation of the PopPK studies conducted on risperidone, eight of them were performed using oral risperidone (either as an oral solution or immediate-release tablet) [43,44,47,48,50,55,56,59], six utilised monthly subcutaneous (SC) risperidone [46,47,51,52,54,57], and five investigated biweekly intramuscular (IM) risperidone [44,45,46,53,54]. On the other hand, among the studies examining paliperidone, three [41,44,48] investigated the oral extended-release (ER) formulation, while five studies assessed IM formulations administered monthly [41,46,57], quarterly [41,49], or biannually [42] IM formulations.

With respect to the injection site for IM formulations, the studies evaluated the administration exclusively in the deltoid [41], the gluteus [42,46], or both the deltoid and gluteus [45,46,49,58]. Notably, in two articles [44,53], no information was provided regarding the injection site. Ten articles included both single-dose and multiple-dose studies [41,43,44,45,49,51,53,54,58,59], while three articles investigated only single-dose [42,46,57] and six articles only multiple-dose [47,48,50,52,55,56] administrations.

On the other hand, 15 articles used liquid chromatography coupled with tandem mass spectrometry (LC-MS/MS) to analyse the samples [41,42,43,46,47,49,50,51,52,53,54,55,57,58,59]. As for the rest of the studies, one used high-performance liquid chromatography with electrochemical detection (HPLC-ECD) [56], and two used radio immunoassay (RIA) [46,59]. It is worth mentioning that two studies [46,59] used more than one bioanalytical method, while in three [44,45,48] articles, no data were found. The LLOQ values ranged from 0.05 ng/mL to 1 ng/mL. However, it is important to note that the maximum value of 1 ng/mL was only reported in two studies [43,56], while in the remaining studies the values were lower, ranging from 0.1 ng/mL to 0.5 ng/mL.

Most models (75.9%) were developed using rich sampling data [41,42,43,44,45,46,47,49,51,53,54,57,59], while only five studies (17.2%) used sparse sampling data [48,50,52,55,56]. In two of the studies (6.9%), no information on the plasma concentration collection times could be obtained [46,58].

All articles used NONMEM for the development of 20 PopPK models for risperidone [43,44,45,46,47,48,50,51,52,53,54,55,56,57,59] and nine for paliperidone [41,42,44,46,48,49,58]. Only three articles developed PopPK models for both risperidone and paliperidone [44,46,48]. In total, 29 PopPK models were developed. Three studies used convolution-based approaches to characterise the absorption phase [41,46,54]. In addition, six studies also included PD modelling [47,48,51,52,54,57].

A total of 15 studies used the first-order conditional estimation (FOCE) with or without interaction as the estimation method [41,42,43,44,45,46,47,49,51,52,53,55,56,57,58], 3 used first order estimation method (FO) [44,50,59], and 1 used stochastic approximation expectation-maximisation (SAEM) [54]. One study did not specify the estimation method used in the analysis [48].

The structural model selected for risperidone in the evaluated studies was either a two-compartment [43,44,47,48,49,51,52,57] or a one-compartment [50,53,55,56] disposition model with first-order elimination. A one-compartment model with first-order elimination adequately described paliperidone concentrations in all studies, except for the study by Korell et al. [44], in which a two-compartment model was selected.

Among the articles that investigated risperidone, 12 considered CYP2D6 [43,44,47,48,50,51,52,53,55,56,57,59] metabolic status as a relevant covariate in the model development, whereas the remaining 3 did not [45,46,54]. Furthermore, none of the studies conducted on paliperidone accounted for CYP2D6 metabolic status [41,42,46,48,49,58].

Six [42,43,45,49,58,59] of the studies applied an additive residual error, another five [41,46,48,55,56] used a proportional model, and eight a mixed residual error model [46,47,50,51,52,53,54,57]. All PopPK models except those developed by Gomeni et al. [41] were evaluated internally, where the most commonly used methods were goodness-of-fit criteria or diagnostics plots and visual predictive check (VPC).

## 4. Discussion

The objective of this systematic review was to identify and synthesise published scientific evidence pertaining to original PopPK models of risperidone and paliperidone. Comprehensive database searches were conducted to minimise publication bias. Furthermore, the review adhered to the PRISMA guidelines to mitigate bias. From our knowledge, this is the first systematic review to summarise the current knowledge of PopPK modelling for risperidone and paliperidone.

When risperidone was administered, PopPK models were typically developed using plasma concentrations of both risperidone and paliperidone, separately. However, for the development of pharmacokinetic-pharmacodynamic (PKPD) models and simulations, the majority of these studies used active moiety plasma concentrations. This was justified by the observation that total active moiety concentration, representing the sum of risperidone and paliperidone concentrations [43,44,45,46,48,53,55,56,59], is considered a more accurate reflection of actual exposure, given their equipotency. In some studies, total active moiety plasma concentrations were calculated as the sum of risperidone and paliperidone plasma concentrations, corrected for the molecular weight difference between risperidone and paliperidone to yield risperidone-equivalent concentrations. This calculation employed the following formula: [risperidone] + [paliperidone] × (410/426) [47,51,52,54,57].

The results indicated that the estimated absorption rate constant (ka) of LAI formulations of risperidone was approximately equal to the elimination rate constant (K_el_), suggestive of “flip-flop” PK. Following oral risperidone administration, some studies implemented flexible absorption models incorporating consecutive zero- and first-order processes and a lag time [44,48,59]. Other studies employed a first-order absorption model [43,47,50,55,56]. In these latter models, limited sampling during the absorption phase precluded precise estimation of the risperidone absorption rate, necessitating the ka value to be fixed to a literature-derived value. SC risperidone administration revealed a complex, multi-phasic absorption profile, characterised by double peaks and a prolonged disposition/elimination phase [47,51,52,57]. These distinct features necessitated the development of complex PK models incorporating dual absorption processes: an initial rapid delivery from the SC injection site, followed by a slower release into the systemic circulation [47,51,52,57]. For IM injection, a combination of zero- and first-order absorption processes adequately described the relatively complex absorption profile [45,53]. Following paliperidone administration, flip-flop kinetics were also observed, with the apparent half-life being determined by the ka. A parallel zero-/first-order absorption model best described the PK of paliperidone after oral or IM administration [48,58]. Both absorption processes were described by non-linear functions, with the rapid absorption process expected to exhibit saturation at lower concentrations than the slow absorption process [42,49].

Only four of the analysed oral risperidone models incorporated first-pass hepatic metabolism into their PK models [44,55,56,59]. Approximately 34% of an oral risperidone dose undergoes pre-systemic conversion to paliperidone, with the remaining 66% reaching systemic circulation as risperidone [56].

The complex, multi-phasic in vivo drug release process poses a significant challenge for effective PK modelling of LAI formulations. Recently, a convolution-based modelling approach demonstrated its utility and flexibility in representing the complex PK of extended-release and LAI products [41,46,54]. This approach employs a piecewise linear approximation of the non-linear input function used to model drug release rate. The PK characteristics of a drug released from an LAI formulation are significantly influenced by its physicochemical properties (solubility and stability), dose, local absorption characteristics at the injection site, injection volume [60], and the physiological properties governing drug diffusion from the administration site into systemic circulation [46]. Samtani et al. [58] observed higher paliperidone plasma concentrations following deltoid muscle injection compared with gluteal muscle injection during paliperidone treatment initiation. Consequently, deltoid muscle injection is recommended for initiating paliperidone treatment. Following multiple injections, the differences between gluteal and deltoid injection sites become less pronounced. Furthermore, the area under the concentration-time curve (AUC) values after deltoid and gluteal injections were comparable, indicating similar overall paliperidone exposure after IM administration of paliperidone at either site. Needle length also influences paliperidone administration; the difference in peak concentration between deltoid and gluteal injections was greater when a 1.5-inch needle was used for deltoid injection compared with a 1-inch needle [58].

Peak plasma concentrations and elimination half-lives were generally similar in paediatric patients [61] to those reported in adults [62]. Thyssen et al. [43] applied allometric scaling to clearances (CL), given the wide range of ages and body weights within their dataset. Using the allometric scaling model for CL, the apparent total CL in a 9-year-old child weighing 29 kg was estimated to be 3.8 L/h, comparable to previous predictions. Renal CL accounted for 20% of total CL in paediatric patients, confirming the renal excretion of the active moiety metabolite, paliperidone [43]. However, given the limited number of published studies in paediatric populations [43,44], further PopPK modelling in this population is warranted to validate these findings.

Risperidone undergoes extensive metabolism to paliperidone [62]. CYP2D6 activity plays a key role in risperidone disposition, leading to lower risperidone and higher paliperidone concentrations in individuals with high CYP2D6 activity [56,63,64,65]. Over 80 allelic variants of the CYP2D6 gene have been identified across different ethnic populations [66], resulting in variable enzymatic activity [67,68]. Feng et al. [50] incorporated a mixture PopPK model to estimate risperidone elimination separately in CYP2D6 polymorphism-related subpopulations: poor metabolisers, intermediate metabolisers, and extensive metabolisers. The relative CLs reflecting risperidone-to-paliperidone conversion were 39 L/h (extensive metabolisers), 36 L/h (intermediate metabolisers), and 12.3 L/h (poor metabolisers). The respective fractions of total risperidone CL attributable to these values were 0.6, 1, and 0.96, respectively. While some studies reported no difference in steady-state active moiety plasma concentrations between CYP2D6 genotypes [64,65,69,70], others observed a 27% increase in active moiety plasma concentration in poor metabolisers [55,63]. Indeed, poor metabolisers exhibited a 3.1-fold increased risk of moderate to severe adverse drug reactions [71]. Discrepancies in findings may be attributable to small sample sizes and limited numbers of poor metaboliser patients [55]. Therefore, these findings require further evaluation in larger clinical trials. Furthermore, the influence of CYP2D6 inhibition should be considered, as one study [72] observed a 4% and 19% decrease in the first-pass effect upon co-administration of weak (methadone, citalopram, duloxetine, venlafaxine, and sertraline) and strong (levomepromazine, haloperidol, paroxetine, or fluoxetine) CYP2D6 inhibitors, respectively.

While risperidone is primarily metabolised to paliperidone by CYP2D6, other alternative metabolic pathways exist, involving other cytochrome P450 isoenzymes, including CYP3A4 [73]. Paliperidone undergoes further metabolism and is also excreted unchanged renally. Although several studies suggest a limited role for CYP3A4 in risperidone metabolism under normal physiological conditions [55,56,59], co-administration with carbamazepine, a CYP3A4 inducer, has been shown to increase risperidone CL, resulting in decreased risperidone and active moiety concentrations [59]. Further research is required to elucidate the mechanisms underlying PK interactions between benzodiazepines and risperidone, as well as to evaluate their clinical significance. Additionally, Thyssen et al. [43] reported that combined P-glycoprotein (P-gp)/CYP3A4 inducers affected the F of the active moiety. However, this finding should be interpreted cautiously, as this combination was used by less than 1% of subjects (7/780: one adolescent and six adults) co-administered with risperidone, and P-gp inhibitors alone did not appear to influence the PK of risperidone or the active moiety [43].

Six studies demonstrated a decrease in CL with decreasing CL_CR_ [42,43,44,49,56,58]. Following oral paliperidone administration, total paliperidone CL was reduced in subjects with renal impairment by 32% in mild, 64% in moderate, and 71% in severe impairment [74]. Thus, PopPK models can be valuable tools for informing dose recommendations in patients with renal dysfunction.

BMI was identified as a statistically significant covariate influencing the magnitude of the peaks in the risperidone and paliperidone concentration-time profiles. Patients with lower BMIs exhibited higher peak concentrations. Increasing BMI from 20 to 33 kg/m^2^ reduced the peak by approximately 80% for both risperidone and paliperidone [47,51,57]. Two PK models incorporating allometric scaling (based on weight) for volume of distribution (Vd) and CL demonstrated superior performance compared to a model without allometric scaling and were therefore used as the reference models for subsequent analysis. However, exploratory analysis revealed collinearity between weight and age, and between weight and CL_CR_ [43,54].

Sex was identified as a significant covariate influencing risperidone and paliperidone CL, ka, F, and Vd in some studies [45,48,53,58]. These observed differences in CL may be related to reported sex-related differences in CYP2D6 activity [50]. Furthermore, Samtani et al. [58] observed decreased ka and F values in females, potentially indicating slightly slower absorption. This may be attributable to differing adipose tissue distribution patterns between sexes. This slower absorption may be offset by a smaller Vd for risperidone in females. Consequently, the overall influence of sex on risperidone and paliperidone PK may be negligible [58].

Dopamine D2 receptor occupancy is a key driver of the clinical efficacy and safety of antipsychotic drugs. The prevailing hypothesis suggests that striatal D2 receptor occupancy should be maintained between 65% and 80% for optimal antipsychotic effect with minimised side effects. A model linking circulating active moiety concentration to D2 receptor occupancy was developed using published data from LAI risperidone studies [57]. Simulated D2 receptor occupancy following repeated monthly doses of 90 mg and 120 mg fell within the 60–80% range. These data, combined with exposure–response analysis of adverse event incidence, suggested that the optimal clinical dose of risperidone lies between 90 mg and 120 mg monthly [57].

Two studies explored and established exposure–response relationships between total active moiety exposure and Positive and Negative Syndrome Scale (PANSS) scores [48,52], with one also evaluating the relationship with the Clinical Global Impression-Severity (CGI-S) score [52]. Pilla et al. [48] concluded that higher observed PANSS scores were associated with an increased dropout likelihood. High dropout rates are a common occurrence in antipsychotic clinical trials, typically ranging from 40 to 70% in placebo groups [75]. Ignoring this information can lead to biased interpretation of the study results. However, a potential limitation of these exposure–response models is the assumption of an instantaneous relationship between drug effect and total active moiety plasma concentration. While evidence supports a rapid onset of antipsychotic effects, achieving full therapeutic effect, particularly regarding negative symptoms, typically requires several weeks.

Preliminary exploratory analysis suggested a correlation between increasing active moiety exposure and the incidence of gastrointestinal disorders [57]. This was confirmed by logistic regression analysis, which identified peak plasma concentration (Cmax) as a statistically significant predictor (*p* < 0.05) of the occurrence of gastrointestinal disorder side effects. Mean total active moiety Cmax values were 25.8 ng/mL and 42 ng/mL at doses of 90 mg and 120 mg monthly, respectively. At these exposure levels, the estimated probability of gastrointestinal disorders was approximately 20% and 40% for the 90 mg and 120 mg risperidone doses, respectively.

Several studies have investigated the relationship between neurological symptoms and plasma concentrations of the active moiety, yielding both positive [32,56,76,77] and negative [68,78,79] findings. In the study by Vandenberghe et al. [55], active moiety minimum plasma concentration (Cmin) was significantly associated with akathisia and tremor, combined with rigidity. Interestingly, a previously published PopPK study did not find an association between akathisia and average plasma concentration of the active moiety [56]. Paliperidone Cmin was not associated with any neurological side effects, potentially due to its lower affinity for D2 receptors and higher affinity for 5-HT2A receptors compared with risperidone [80]. This observation is consistent with prospective studies reporting a reduction in neurological symptoms after switching from risperidone to paliperidone [81,82]. Logistic regression analysis indicated that values of active moiety Cmin exceeding 40 ng/mL are associated with a risk of developing neurological symptoms greater than 70% [55]. Given that 40 ng/mL represents the median value of the proposed therapeutic range (20–60 ng/mL) [83], this suggests that the upper limit of this range should only be targeted in cases of insufficient or absent therapeutic response.

In contrast, prolactin concentration in women was associated with paliperidone Cmin. Paliperidone’s longer half-life and greater hydrophilicity compared to risperidone are noteworthy, considering the pituitary gland’s location outside the blood–brain barrier [84,85,86,87]. Oestrogens promote an increase in pituitary lactotrophic cells and a reduction in D2 receptor synthesis [88], potentially conferring greater sensitivity to prolactin release induced by D2 receptor antagonists in women [89].

## 5. Limitations

The studies included in this analysis comprised only patients with normal renal function, with calculated CL_CR_ over 70 mL/min. A previous PopPK study of risperidone and the active moiety in patients with dementia revealed decreased apparent active moiety CL with CL_CR_ values below 50 mL/min, associated with advanced age. In trials conducted in patients with bipolar disorder, only a small proportion of patients were older than 60, with CL_CR_ values below 50 mL/min. This limited representation precluded the identification of a CL_CR_ effect on paliperidone CL and active moiety quasi-clearance [59].

While numerous PopPK models of risperidone and paliperidone have been published in adults, limited external evaluation of these models has been conducted. This has hindered the implementation of Bayesian-guided risperidone and paliperidone dosing in routine clinical practice. Evaluating models using datasets independent of the model-building process allows for the assessment of model generalisability, a crucial factor when selecting a model for clinical application.

## 6. Conclusions

Numerous PopPK models for risperidone and paliperidone have been published. The models differ highly on their absorption model structure and the significant covariates included. A comprehensive and systematic external evaluation of these models is essential to assess their generalisability and facilitate the implementation of accurate and reliable Bayesian-guided dosing decisions across diverse patient populations. Future risperidone and paliperidone PopPK model development should consider the impact of clinically relevant drug–drug interactions, as well as the influence of different risperidone and paliperidone formulations and brands.

## Figures and Tables

**Table 1:** Summary of the population characteristics of the included studies.

Authors (Year)	Country	No. of Subjects (Male/Female)	No. Observations	Sampling Design	Age Mean ± SD	BMI Mean ± SD	Weight (kg) Mean ± SD	CLcr (mL/min) Mean ± SD	Formulation	Daily Dose (mg) Median [range]	Bioanalytical Method (LLOQ (ng/mL))	Pk Model Summary/ Influence CYP2D6 Status on the Model
Risperidone												
Vermeulen A et al. (2007) [42]	NA	407 (275/132)	5359	RS	NA	NA	NA	NA	tablet	[2–8]	RIA and LC-MS/MS (0.10)	two compartments Yes
Feng Y et al. (2008) [43]	MC	490 (331/159)	1236	SS	49.1 ± 18.8	NA	84.1 ± 22.5	NA	tablet	[0.5–6]	LC–MS/MS (0.1)	one compartment Yes
Thyssen A et al. (2010) [44]	MC	780 (469/311) ^a^	4134	RS	28.1 ± 16.6 *	NA	74.3 ± 34.5 *	149.8 ± 67.5 *	tablet or oral solution	oral solution: [0.35–6] tablet: [0.25–15]	LC–MS/MS (0.10–1.0)	two compartments Yes
Locatelli I et al. (2010) [45]	Slovenia	50 (39/11)	296	SS	34.0 ± 11.0 *	NA	75.7 ± 14.0 *	117.5 ± 33.5 *	tablet	one daily: 3 [2–4] two daily: 4 [1.5–6]	HPLC-ECD (1.0)	one compartment Yes
Pilla V et al. (2013) [46]	MC	1471 (NA)	NA	RS	NA	NA	NA	NA	tablet	[0.5–8]	NA	two compartments Yes
Gomeni R et al. (2013) [47]	EEUU	45 (32/13)	NA	RS	43.0 ± 1.6	28.4 ± 0.5	88.7 ± 2.0	NA	SC injection (monthly)	[60–120]	LC–MS/MS (0.1)	two compartments No
Laffont C et al. (2014) [48]	MC	45 (33/12)	5232	RS	42.6 ± 9.2	28.6 ± 4.3	86.8 ± 15.0	NA	SC injection (monthly)/tablet	[60–120] and [2–4]	LC–MS/MS (0.1)	oral: one compartment LAI: two compartments No
Laffont C et al. (2015) [49]	MC	90 (65/25)	7568	RS	42.8 ± 9.8	28.5 ± 3.9	87.8 ± 14.3	NA	SC injection (monthly)	[60–120]	LC–MS/MS (0.1)	two compartments No
Vandenberghe F et al. (2015) [50]	Switzerland	150 (82/68)	178	SS	32.5 ± 17.7 *	25.9 ± 6.3 *	NA	115 ± 36 *	tablet	[0.5–8]	LC–MS/MS (NA)	one compartment Yes
Ivaturi V et al. (2017) [51]	MC	225 ^b^ (177/48)	3154	SS	40.4 ± 9.4	29.5 ± 6.4	89.7 ± 19.6	122.3 ± 36.3	SC injection (monthly)	[90–120]	LC–MS/MS (0.1)	two compartments Yes
Korell J et al. (2017) [52]	MC	NA	NA	RS	NA	NA	NA	NA	tablet	NA	NA	two compartments Yes
Korell J et al. (2017) [52]	MC	133	3051	RS	NA	NA	NA	NA	IM injection	NA	NA	one compartment No
Wang W et al. (2024) [53]	MC	102 (77/25)	2216	RS	46.8 ± 9.6	28.8 ± 4.7	87.5 ± 15.7	NA	IM injection ^c^ (weekly)	[12.5–50]	LC–MS/MS (0.05)	one compartment No
Wang W et al. (2024) [53]	MC	69 (51/18)	1766	RS	47.1 ± 9.9	28.7 ± 4.6	87.9 ± 16.4	NA	IM injection ^c^ (weekly)	[25–50]	LC–MS/MS (0.05)	one compartment No
Laveille C et al. (2024) [54]	MC	447 (316/131)	6288	RS	42.0 ±11.8 *	29.3 ± 6.4 *	NA	140 ± 57 *	IM injection (monthly)	[25–100]	NA	one compartment No
Gomeni R et al. (2021) [55]	NA	26 (16/10)	NA	RS	45.0 ± 10.0	NA	NA	NA	IM injection (weekly)	50	RIA (0.2)	one compartment No
Gomeni R et al. (2021) [55]	NA	NA	NA	NA	NA	NA	NA	NA	SC injection (monthly)	[60–120]	NA (NA)	two compartments No
Perlstein I et al. (2022) [56]	MC	99 (79/20)	NA	RS	44.4 ± 8.8	28.6 ± 4.4	NA	116 ± 22	SC injection of (monthly)	[75–225]	LC–MS/MS (risperidone: 0.10; paliperidone: 0.292)	two compartments No
Paliperidone												
Samtani M et al. (2009) [57]	NA	1401 (919/482)	15754	NA	44.5 ± 14.8 *	34.3 ± 13.5 *	NA	182 ± 114 *	IM injection PP1M	[25–150]	LC–MS/MS (NA)	one compartment No
Pilla V et al. (2013) [46]	MC	948 (NA)	NA	SS	NA	NA	NA	NA	tablet	[3–15]	NA	
Magnusson M et al. (2017) [58]	MC	651 (463/188)	8990	RS	40.3 ± 12.5 *	27.3 ± 5.8 *	NA	124 ± 47 *	IM injection of PP3M	[175–525]	LC-MS/MS (0.1)	one compartment No
Korell J et al. (2017) [52]	MC	327 (NA)	NA	RS	NA	NA	NA	NA	table	NA	NA	two compartments No
T’jollyn H et al. (2024) [59]	MC	477 (326/151)	10,784	RS	41.2 ± 11.8	27.9 ± 5.0	81.9 ± 16.9	NA	IM injection of PP6M	[700–1000]	LC–MS/MS (0.2)	one compartment No
Gomeni R et al. (2021) [55]	MC	51 ((36/15)	NA	RS	40.3 ± 11.5	28.4 ± 4.0	86.4 ± 14.4	NA	IM injection of PP1M	[25–150]	LC–MS/MS (0.1)	one compartment No
Gomeni R et al. (2023) [60]	MC	NA	NA	RS	NA	NA	NA	NA	tablet	[3–15]	LC–MS/MS (NA)	one compartment No
Gomeni R et al. (2023) [60]	MC	NA	NA	RS	NA	NA	NA	NA	IM injection of PP1M	[25–150]	LC–MS/MS (0.2)	one compartment No
Gomeni R et al. (2023) [60]	MC	NA	NA	RS	NA	NA	NA	NA	PP3M	[175–525]	LC–MS/MS (0.1)	one compartment No

^a^: 52 children (34/18), 252 adolescents (141/111), 476 adults (294/182). ^b^: 102 Rykindo (77/25) and 69 Consta (51/18). ^c^: different formulations. *: parameters estimated from the median and range. BMI: body mass index; CLcr: creatinine clearance; IM: intramuscular; HPLC-ECD: high-performance chromatography with electrochemical detection; LC-MS/MS: liquid chromatography–tandem mass spectrometry; LLOQ: lower limit of quantification; MC: multicentre; NA: not available; PP1M: paliperidone palmitate monthly; PP3M: paliperidone palmitate quarterly; PP6M: paliperidone palmitate half-yearly; RIA: radio immunoassay; RS: rich sampling; SC: subcutaneous; SD; standard deviation; SS: sparse sampling.

**Table 2:** Summary of the PopPK parameters of the developed models included in each study.

Authors (Year)	Estimation Method	Fixed Effect Parameters	Between-Subject Variability	Residual Unexplained Variability	Internal Validation	Simulation Target
Risperidone						
Vermeulen A et al. (2007) [42]	FO	ka = 2.34 h^−1^; CL_RISP_ = 2.84 L/h; CL_RISP_CBZ_ = 6.22 L/h; CL_PAL_ = 5.99 L/h; CL_PAL_CBZ_ = 6.22 L/h; CL_f,PM_ = 1.18 L/h; CL_f,IM_ = 4.37 L/h; CL_f,EM_ = 19.6 L/h; Q_RISP_ = 3.65 L/h; Q_PAL_ = 1.67 L/h; V_C,RISP_ = 137 L; V_P,RISP_ = 100 L; V_C,PAL_ = 137 L; V_P,PAL_ = 91.8 L; tlag = 0.165 h; D = 0.458 h; F = 100%; FP_PM_ = 1.75%; FP_IM_ = 10.9%; FP_EM_ = 41.3%	ka = 149% CL_RISP_ = 184% CL_f_ = 33.3% Q_RISP_ = 215% CL_PAL_ = 20.4% V_C,RISP_ = 30% V_P,RISP_ = 53.9% V_C,PAL_ = 30% V_P,PAL_ = 80.7% tlag = 41% D = 113% FP = 117%	RISP: 54.9% PAL: 60.2%	VPC, GOF diagnostic plots	No
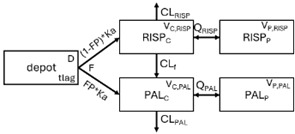						
Feng Y et al. (2008) [43]	FO	ka (fx) = 1.7 h^−1^; CL_RISP,PM_ = 12.9 L/h; CL_RISP,EM_ = 65.4 L/h; CL_RISP,IM_ = 36 L/h; CL_PAL_ = 8.83 × $e^{-0.378\times (\frac{age}{49.1})}$ L/h; V_C,RISP//PAL_ = 444 L; kr_CONV_,_PM_ = $\frac{CL\times 0.961}{Vc}$ h^−1^; kr_CONV_,_EM_ = $\frac{CL\times 0.595}{Vc}$ h^−1^; kr_CONV_,_IM_ = $\frac{Cl\times 1}{Vc}$ h^.1^	ka = 53.7% CL_RISP,PM_ = 95.9% CL_RISP,EM_ = 56.6% V_C, RISP//PAL_ = 36.1%	RISP: 63.9%/4.29 mg/mL PAL: 37.9%/0.88 mg/mL	GOF diagnostic plots	No
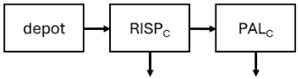						
Thyssen A et al. (2010) [44]	FOCE	Active moiety: ka = 2.39 h^−1^; CL = (4.66 × ${\left(\frac{WT}{70}\right)}^{0.75}$ + 0.00831 × CLcr + 0.862 (if race black) × ${\left(\frac{age}{18.1}\right)}^{-0.172}\;$L/h; Q = 1.35 L/h; tlag = 0.235 h; V_C_ = (137 − 40.3 (if study 3,6,7,8)) × ($\frac{WT}{70}$) L; V_P_ = 86.8 × ($\frac{WT}{70}$) L; F = 100–46.7% (if coadministered with P-gp inducer) RISP: ka_study1–5,9_ = 0.84 h^−1^; ka_study6–8_ = 2.53 h^−1^; CL = 32.2 × ${\left(\frac{WT}{70}\right)}^{0.75}$ × $0.324^{0.189}$ (if PM) L/h; Q = 3.24 L/h; V_C_ = 142 × ($\frac{WT}{70}$) L; V_P_ = 175 × ($\frac{WT}{70}$) L; tlag = 0.223 h; F_PM_ = 123%; F_EM_ (fx) = 100%	CL = 24.2% F = 32.4% CL = 13.7% V_p_ = 29.9% F = 70.5%	Study_6–8_: 0.270 mg/mL Study_1–5,9_: 0.186 mg/mL Study_6–8_: 0.714 mg/mL Study_1–5,9_: 0.301 mg/mL	GOF diagnostic plots	Monte-Carlo simulations of Cp-time for different dosing regimens in different age groups
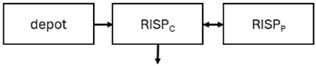						
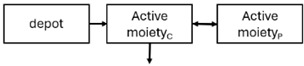						
Locatelli I et al. (2010) [45]	FOCE	ka (fix) = 2.43 h^−1^; CL_PAL_ = 8.45 × $\left(\frac{CLcr}{WT}\right)$^0.466^ × 0.648^MZ^ × 1.47^DZ^ L/h; CL_f(−)_ = 9.74 × 0.118^PM^ × 0.301^IM^ × 0.713^EM1^ × 0.902^EM2^ × 1.19^UM^ × 0.432^MZ^ L/h; CL_f(+)_ = 15.5 × 0.0813^PM^ × 0.285^IM^ × 0.554^EM1^ × 0.941^EM2^ × 0.942^UM^ L/h; Q_PAL(+) − (−)_ = 6.01 L/h; V = 2.05 × WT L; FP = $\frac{2\times 0.379\times (CLf(-)+CLf(+))}{9.74+15.5+CLf(-)+CLf(+)}$%; FP_(+)_ = FP × $\frac{CLf(+)}{CLf(-)+CLf(+)}$%; FP_(−)_ = FP × $\frac{CLf(-)}{CLf(-)+CLf(+)}$%	CL_PAL_ = 21.1% CL_f(−)_ = 49.5% CL_f(+)_ = 43.5%	RISP: 35.9% PAL (−): 18.5% PAL (+): 27.4%	bootstrap, NPC, GOF diagnostic criteria	Cp-time grouped by status CYP2D6 of RISP, active moiety, and enantiomers in a typical patient (70 kg and 120 mL/min creatinine clearance) with 2 mg twice daily
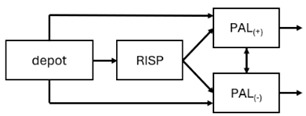						
Pilla V et al. (2013) [46]	NA	ka = 2.37 h^−1^; CL = 2.57 L/h; CL_PM_ = 0.44 L/h; CL_IM_ = 2.81 L/h; CL_EM_ = 18.4 L/h; Q = 3.8 L/h; V_c_ = 144 L; V_p_ = 101 L; tlag = 0.16 h; D = 0.47 h	CL = 169% V_C_ = 54%	RISP: 54.8% PAL: 63.3%	Bootstrap, Monte-Carlo simulations and VPC	No
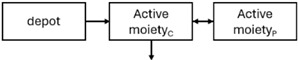						
Gomeni R et al. (2013) [47]	FOCE-I	ka_rapid_ = 0.60 × $e^{-0.16\times BMI}$ h^−1^; ka_slow_ = 0.24 × $e^{-0.068\times BMI}$ h^−1^; ktr = 0.03 h^−1^; kr_C-P_ = 0.59 h^−1^; kr_P-C_ = 0.01 h^−1^; kr_CONV_ = 0.10 h^−1^; V_RISP_ = 1780 × $e^{-0.007\times DOSE}$ L; V_PAL_ = 190 × $e^{-0.007\times DOSE}$ L; k_el,RISP_ = 0.03 h^−1^; k_el,PAL_ = 0.07 h^−1^	ka_rapid_ = 50% ka_slow_ = 34.6% ktr = 40.0% kr_C-P_ = 72.1% kr_P-C_ = 49.0% kr_CONV_ = 75.5% k_el,PAL_ = 33.2% V_PAL_ = 26.5% k_el,RISP_ = 42.4%	0.34 mg/L 26.5%	Bootstrap, VPC and GOF criteria	Probability of gastrointestinal adverse events-Cp_max_
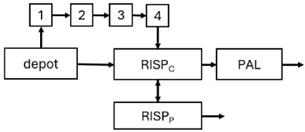						
Laffont C et al. (2014) [48]	FOCE-I	Oral RISP: ka = 3.64 h^−1^; kr_CONV_ = 0.0990 h^−1^; V_PAL_ = 63.8 L; V_RISP_ = V_PAL_ × 3.18 L; k_el,RISP_ = 0.0344 h^−1^; k_el,PAL_ = 0.0782 h^−1^ RISP monthly: ka_rapid_ = 0.0266 × $e^{-00.0425\times BMI}$ h^−1^; ka_slow_ = 0.0185 h^−1^; ktr = 0.0247 h^−1^; kr_C-P_ = 0.572 h^−1^; kr_P-C_ = 0.0114 h^−1^; kr_CONV_ = 0.0474 h^−1^; V_PAL_ = 171 L; V_RISP_ = V_PAL_ × 1.34 L; k_el,RISP_ = 0.011 h^−1^; k_el,PAL_ = 0.0484 h^−1^	k_el,RISP_ = 64% kr_CONV_ = 87% V_PAL_ = 37% kr_el,PAL_ = 15% ka_rapid_ = 31% ka_slow_ = 58% ktr = 40% kr_C-P_ = 49% kr_P-C_ = 89% kr_CONV_ = 63% k_el,PAL_ = 14% V_PAL_ = 35%	64.0% 0.089 mg/L 57.4%	GOF diagnostic plots and VPC	Dopamine D2 receptor occupancy-time of different doses, Cp-time profiles of different doses
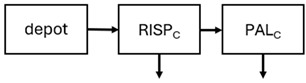						
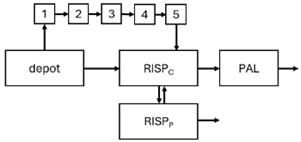						
Laffont C et al. (2015) [49]	FOCE-I	ka_rapid_ = 0.0151 × $e^{-0.0803\times (BMI-29.2)}$ h^−1^; ka_slow blacks_ = 0. 0513 h^−1^; ka_slow_ = 0.0258 h^−1^; ktr = 0.0283 h^−1^; kr_C-P_ = 0.537 h^−1^; kr_P-C_ = 0.0226 h^−1^; kr_CONV_ = 0.0474 h^−1^; V_PAL_ = 125 L; V_RISP_ = V_PAL_ × 3.65 L; k_el,RISP_ = 0.0092 h^−1^; k_el,PAL_ = 0.0620 h^−1^	ka_rapid_ = 44% ka_slow blacks_ = 43% ka_slow_ = 43% k_el,RISP_ = 91% ktr = 46% kr_C-P_ = 57% kr_P-C_ = 59% kr_CONV_ = 66% k_el,PAL_ = 27% V_PAL_ = 27%	0.0774 ng/L 57.9%	GOF diagnostic plots and VPC	Cp-time profiles of different doses of RISP and PP1M; dopamine D2 receptor occupancy-time of different doses of RISP and PP1M
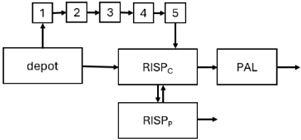						
Vandenberghe F et al. (2015) [50]	FOCE-I	ka_1_ = 3.1 × (1-FP) h^−1^; ka_2_ = 3.1 × FP h^−1^; ka (fix) = 3.1 h^−1^; CL_RISP_ = 4.6 L/h; CL_PAL_ = 6.1 × (1 − 0.26 × $(\frac{age-37.3}{37.3}$) L/h; kr_CONV_ = 4.9 h^−1^; V = 250 L; FP = 93.1; FP_PM_ = 88.1%; FP_IM_ = 92.25% FP_MI_ = 92.59%; FP_SI_ = 91.6%	CL_RISP_ = 41% CL_PAL_ = 32% Logit_FP_ = 132%	RISP: 41.0% PAL: 37.0%	goodness-of-fit plots, bootstrap, NPDE y VPC	AUC-status CYP2D6 for RISP, PAL, and the active moiety
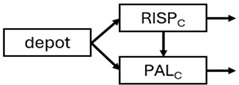						
Ivaturi V et al. (2017) [51]	FOCE-I	ka_rapid_ = 0.005 h^−1^; ka_slow_ = 0.016 h^−1^; V_RISP_, V_PAL_ = 129 L; ktr = 0.023 h^−1^; kr_C-P_ = 0.841 h^−1^; kr_P-C_ = 0.006 h^−1^; kr_CONV_ = 0.221 × (1 − 0.76 (if IM)) × (1–0.942 (if PM)) h^−1^; k_el,RISP_ = 0.043 h^−1^; k_el,PAL_ = 0.069 h^−1^	ka_rapid_ = 42% ka_slow_ = 32% ktr = 42% kr_C-P_ = 45% kr_P-C_ = 68% kr_CONV_ = 49% k_el,PAL_ = 19% V_RISP_, V_PAL_ = 39%	0.137 mg/L 29.7%	Bootstrap and pcVPC	No
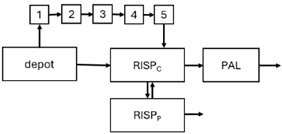						
Korell J et al. (2017) ^a^ [52]	FO	ka = 2.01 h^−1^; CL_RISP_ = 5.95 × ($\frac{AGE}{23.2})$^−0.769^ L/h; CL_RISP_KNOWN_CBZ_ = 12.1 L/h; CL_RISP_UNKNOWN_CBZ_ = 5.63 L/h; CL_f,PM_ = 1.47 L/h; CL_f,IM_ = 9.01 L/h; CL_f,EM_ = 17.5 L/h; Q_RISP_ = 2.67 L/h; Q_PAL_ = 1.54 L/h; CL_PAL_KNOWN_CBZ_ = 5.50 L/h; CL_PAL_UNKNOWN_CBZ_ = 4.94 L/h; V_C,PAL_, V_C,RISP_ = 113 L; V_P,RISP_ = 71.9 L; V_P,PAL_ = 83.3 L; tlag = 0.168 h; D = 0.447 h; FP__PM_ = 3.69%; FP__IM_ = 7.10%; FP__EM_ = 42.7%	ka = 91.4% CL_f_ = 42.9 Q_RISP_ = 78% Q_PAL_ = 0 (fix) CL_PAL_ = 16.5% V_C,PAL&RISP_ = 21% V_P,RISP_ = 50% V_P,PAL_ = 56.7% tlag = 38.1% D = 71.3% FP = 102%	RISP: 29.5% PAL: 31.1%	GOF, VPC	Range of reference
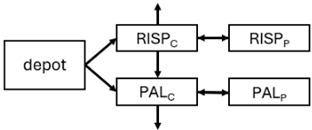						
Korell J et al. (2017) ^a^ [52]	FOCE	CL = 4.27 × $\left(\frac{LBW}{50}\right)$^0.725^ L/h; V_c_ = 351 L; ktr_1_ (fix) = 100 h^−1^; ktr_2_ = 0.0073 h^−1^; ktr_3_ = 0.0177 h^−1^; F_1_ = 3.21%; F_2_ = 21.7 × 3.56 (if B3)%; F_3_ = 75.09%; tlag_2_ (fix) = 0; tlag_3_ = 613 h	CL = 39.1% ktr_2_ = 66.7% F_1_ = 32.1% (B3) F_2_ = 82.4% (B1), 46.4% (B3)	B1: 28.4% B3: 47.4%	GOF, VPC	Range of reference
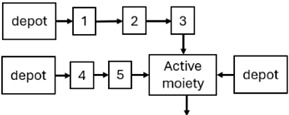						
Wang W et al. (2024) [53]	FOCE-I	RISP weekly ^c^ ka_1study 1S01 and 104_ = 0.012 h^−1^; ka_1study 102_ = 0.016 h^−1^; CL_male_ = 7.78 L/h; ka_2_ = 0.0049 h^−1^; CL_female_ = 6.39 L/h; D_2_ = 18.336 h; D_3_ = 52.32 h; F_1_ = 43%; F_2_ = 14.8%; F_3_ = 42.2%; tlag = 319.2 h; tlag_3_ = 83.28 h RISP weekly ^c^: ka_study 104_ = 0.0075 h^−1^; ka_study 102_ = 0.011 h^−1^; ka_2_ = 0.0035 L/h; CL_male_ = 4.5 L/h; D_2_ = 1.12 h; D_3_ = 573.6 h; F_1_ = 75.5%; F_2_ = 11.9%; F_3_ = 12.6%; tlag = 648 h; tlag_3_ = 1.0008 h	ka_1study 1S01 and 104_ = 27% CL_male_ = 35% D_2_ = 52% F_1_ = 52% F_2_ = 57% ka_1_ = 26% CL_male_ = 34% tlag = 93.2%	26.0% 0.636 mg/L 3.3% 74.2 mg/L	VPC, goodness-of-fit diagnostics plots, bootstrap	Cp-time profiles at steady state for active moiety, for formulation switch, for dosing windows, and for oral supplementary treatment
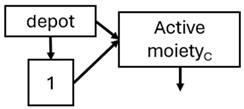						
Laveille C et al. (2024) [54]	FOCE-I	ka_1_ = 0.00583 h^−1^; ka_2_ = 0.000691 h^−1^; ka_3_ = 0.00763 h^−1^; ka_5_ = 2 h^−1^ CL = 4.67 × $\left(\frac{BMI}{28}\right)$^−0.267^ × (1–0.188 (if female)) L/h; V = 248 L; tlag_2_, tlag_3_ = 255 h; D = 308 h; F_1_ = 0.431 × $e^{-0-119}$ (if deltoid injection) × (1–0.0725) × 100%; F_2_ = 0.0873 × (1–0.431 × $e^{-0-119}$ (if deltoid injection)) × 100%; F_3_ = (1–0.431 × $e^{-0-119}$ (if deltoid injection)) × (1–0.0873) × 100%; F_4_ = 0.431 × $e^{-0-119}$ (if deltoid injection) × 0.0725 × 100%; F_5_studyBORIS_ = 0.585; F_5_studyPRISMA-3_ = 1.31	CL = 32.7% V = 34.2% ka_1_ = 16.8% ka_2_ = 109% ka_3_ = 25.1% D = 15.1% F_5_BORIS_ = 25.8% F_5_PRISMA-3_ = 159%	18.2%	GOF and pcVPC	Cp-time profiles of different doses at different INSJ
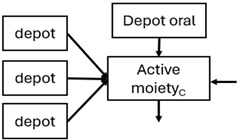						
Gomeni R et al. (2021) ^b^ [55]	FOCE-I	kr_C-P Monthly_ = 0.02 h^−1^; kr_P-C Monthly_ = 0.133 h^−1^; k_el Monthly_ = 0.053 h^−1^; k_el two weekly_ = 0.165 h^−1^	NA	RISP two weekly: 31.3% RISP monthly: 21.9% 0.355 mg/L	GOF criteria	Cp-time of different dosing regimens, for a lead-in oral dosing period and for a discontinuation treatment; %available dose absorbed-time
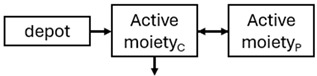						
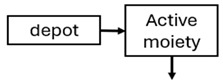						
Perlstein I et al. (2022) [56]	SAEM	CL = 2.1 × ${\left(\frac{WT}{1.70}\right)}^{0.75}$ L/h; V = 374 × ${\left(\frac{WT}{70}\right)}^{1}$ L; kr_C-P_ = 0.008 h^−1^; kr_P-C_ = 0.14 h^.1^	CL = 34.2% V = 38.3% kr_C-P_ = 118.7% kr_P-C_ = 137.8%	38.9% 1.58 mg/L	GOF plots, VPC	Dopamine D2 receptor occupancy-time of different dosing regimens; Cp-time of active moiety for formulation switch and for different dosing regimens
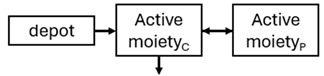						
Paliperidone						
Samtani M et al. (2009) [57]	FOCE	ka = 0.488 × 10^−3^ × 0.765 (if female) × 1.23 (if deltoid injection) × ${\left(\frac{age}{42}\right)}^{0.311}$ × IVOL^−0.359^; CL = 4.95 × ${\left(\frac{CLcr}{110.6}\right)}^{0.376}$; V_C_ = 391 × 0.726 (if female) × ${\left(\frac{BMI}{26.8}\right)}^{0.889}$ h^−1^; tlag/D = 319 h; F_1_ = 16.8 × 0.781 (if female) × 1.37 (if deltoid injection) × 1.54 (if deltoid muscle 1.5-inch needle) × ${\left(\frac{BMI}{26.8}\right)}^{-0.642}$ × IVOL^−0.228^%; F_2_ = 1 − F_1_%	ka = 59% CL = 40% V_C_ = 69% F_1_ = 25.3%	0.22 mg/L	VPC, GOF diagnostic plots	No
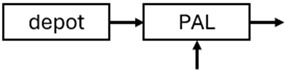						
Pilla V et al. (2013) [46]	NA	ka = 0.57 × h^−1^; CL = 14.1 L/h; V_C_ = 475 L; tlag = 0.67 h; D = 23.6 h	CL = 51%	61.6%	Bootstrap, Monte-Carlo simulations and VPC	No
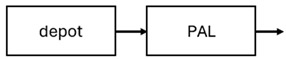						
Magnusson et al. (2017) [58]	FOCE	ka_slow_ = 0.746 (if deltoid injection) × 0.794 (if female) × 90.4 × 10^−3^ × ${\left(\frac{IVOL}{1.75}\right)}^{0.808}$ × $\frac{{A(1)}^{1.44}}{{A(1)}^{1.44}+120^{1.44}\times ({\frac{IVOL}{1.75})}^{0.808}}$ h^−1^; ka_rapid_ = 164 × 10^0.3^ × ${\left(\frac{IVOL}{1.75}\right)}^{0.808}$ × $\frac{A(3)}{A\left(3\right)+21.4\times ({\frac{IVOL}{1.75})}^{0.808}}$ h^−1^; CL = 3.84 × $({\frac{CLcr}{115})}^{0.316}$ L/h; V = 1960 × ${\left(\frac{BMI}{26.15}\right)}^{1.18}\;$L; F_1_ = 20.9%; F_2_ = 1 − F_1_ = 79.1%	Ka_slow max_ 82.7% k_amt1,50_ 50% k_amt3,50_ 86.7% CL = 35.7% V = 62.8% F = 85.4%	30.6%	GOF criteria, pcVPC and VPC	No
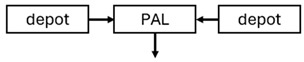						
Korell J et al. (2017) ^a^ [52]	FO	ka = 0.630 h^−1^; CL = 10.9 × $(\frac{WT}{74.4}$)^0.727^ + CLcr × 0.024 L/h; Q = 22.0 L/h; V_C_ = 198 L; V_P_ = 224 L; F (fix) = 100%; tlag = 0.761 h; D = 25 h	ka = 59.9% CL = 44.4% V_C_ = 34.5% V_P_ = 28.6%	20.5%	VPC	Range of reference
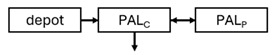						
T’jollyn H et al. (2024) [59]	FOCE	ka_rapid_ = 149 × (${\frac{IVOL}{1.75})}^{0.890}$ × $\frac{A(3)}{A\left(3\right)+23.8\times {\left(\frac{IVOL}{1.75}\right)}^{0.890}}$ h^−1^; ka_slow_ (fx) = 90.4 × (${\frac{IVOL}{1.75})}^{0.890}$ × 0.746 (if gluteal injection) × 0.794 (if women) × $\frac{A(3)}{{A(1)}^{1.44}+120^{1.44}\times {\left(\frac{IVOL}{1.75}\right)}^{0.890}}$ h^−1^; CL = 3.90 × (${\frac{CLcr}{115})}^{0.281}$ L/h; V (fix) = 1960 × (${\frac{BMI}{26.15})}^{1.18}$ L; F (fix) = 20.9%	k_amt3.50_ 88.1% Ka_slow max_ 81.0% k_amt1.50_ 50% CL = 33.5% V = 62.8% F = 85.4%	28.6%	GOF criteria, VPC, NPC	Cp-time profiles compared across sex groups, BMI, RI, age; Cp-time profiles of missed doses, INSJ, different dosing regimens, different transition of formulations, dosing window
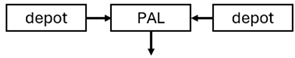						
Gomeni R et al. (2021) ^b^ [55]	FOCE-I	k_el PP1M_ = 0.022 h^−1^	NA	28.8%	GOF criteria	Cp-time of different dosing regimens, for loading dose and discontinuation treatment; %available dose absorbed-time
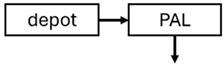						
Gomeni R et al. (2023) [60]	FOCE-I	CL_PAL ER_ = 15.13 L/h; CL_PP1M_ = 5.04 L/h; CL _PP3M_ = 4.38 L/h; V_PAL ER_ = 446; V_PP1M_ = 6080 L; V_PP3M_ = 11,700 L	NA	NA	No	Cp-time for different dosing regimens (switching of formulations, different doses) fraction of dose released-time
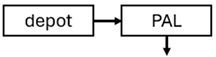						

^a^: same article; ^b^: same article; ^c^: different formulations; (+) and (−): 9-OHrisperidone enantiomers; A: amount; AUC: area under the concentration; B1: formulation batch 1; B3: formulation Batch 3; C: central compartment; CBZ: carbamazepine; CL: clearance (L/h); CLf: apparent clearance from risperidone to 9-OHrisperidone (L/h); CLcr: creatinine clearance; Cp: plasma concentration; D: release duration (h); DZ: diazepam; EM: extensive metabolisers; F: bioavailability (%); FP (relative, %): first-pass metabolism; GOF: goodness of fit; IM: intermediate metabolisers; INSJ: injection site; IVOL: injection volume; ka: absorption rate constant (h^−1^); k_amt1,50_ (mg): the dose amount needed to reach 50% of the maximum absorption capacity for the slow absorption process; k_amt3,50_ (mg): the dose amount needed to reach 50% of the maximum absorption capacity for the rapid absorption process; Ka_rapid,max_ (μg/h): maximum (zero-order) absorption rate for the rapid absorption process; Ka_slow,max_ (μg/h): maximum (zero-order) absorption rate for the slow absorption process; k_el_: elimination constant (h^−1^); KF: fraction of risperidone to 9-OHrisperidone; kr: intercompartmental flow rate constant (h^−1^); kr_CONV_: rate constant conversion risperidone to 9-OHrisperidone (h^−1^); ktr: absorption transit rate constant (h^−1^); LAI: long acting injection; LBW: lean body weight; MI: moderate CYP2D6 inhibitor¸MZ: midazolam; NA: not available; NPC: numerical predictive check; NPDE: normalised prediction distribution errors; P: peripherical compartment; PAL: 9-OHrisperidone; pcVPC: prediction-corrected visual predictive check; P-gp: P-glycoprotein; PM: poor metabolisers; Q: apparent intercompartmental flow (L/h); RI: renal impairment; RISP: risperidone; SI: strong CYP2D6 inhibitor; tlag: time delay in abspsortion (h); UM: ultra rapid metabolisers; V: apparent volume of distribution (L); VPC: visual predictive check; WT: weight.

## Data Availability

Data sharing is not applicable.

## Supplementary Materials

The following supporting information can be downloaded at: https://www.mdpi.com/article/10.3390/ph18050698/s1, Table S1: PRISMA 2020 Checklist; Table S2: Search strategies.

## Abbreviations

The following abbreviations are used in this manuscript:

A	amount
AUC	concentration-time curve
B1	formulation Batch 3
B3	formulation Batch 3
BIREME	Latin American and Caribbean Center on Health Sciences Information
BMI	body mass index
C	central
CBZ	carbamazepine
CGI-S	Clinical Global Impression-Severity
CL	clearance
CLf	apparent clearance from risperidone to 9-OHrisperidone
CLcr	creatinine clearance
Cmax	maximum plasma concentration
Cmin	minimum plasma concentration
Cp	plasma concentration
CV	coefficient of variation
D	release duration
DZ	diazepam
DeCS	Health Sciences Descriptors
EM	extensive metaboliser
ER	extended-release
F	bioavailability
FO	first-order estimation method
FP	first-pass metabolism
FOCE	first-order conditional estimation
GOF	goodness of fit
HPLC-ECD	high-performance liquid chromatography with electrochemical detection
IIV	inter-individual variability
IM	intramuscular
INSJ	injection site
IOV	inter-occasion variability
IVOL	injection volume
ka	absorption rate constant
kamt1,50	dose amount needed to reach 50% of the maximum absorption capacity for the slow absorption process
kamt3,50	dose amount needed to reach 50% of the maximum absorption capacity for the rapid absorption process
Karapid,max	maximum absorption rate for the rapid absorption process
Kaslow,max	maximum absorption rate for the slow absorption process
k_el_	elimination rate constant
KF	fraction of risperidone to 9-OHrisperidone
kr	intercompartmental flow rate constant
kr_conv_	rate constant conversion riperidone to 9-OHrisperidone
ktr	absorption transit rate constant
LAI	long-acting injectable
LC-MS/MS	liquid chromatography coupled with tandem mass spectrometry
LILACS	Latin American and Caribbean Health Sciences Literature
LLOQ	lower limit of quantification
MC	multicentre
MeSH	Medical Subject Headings
MI	moderate CYP2D6 inhibitor
MIDZ	midazolam
MIPD	model-informed precision dosing
NA	not available
NPC	numerical predictive check
NPDE	normalised prediction distribution errors
P	peripheral compartment
PAL	9-OHrisperidone
PANSS	Positive and Negative Syndrome Scale
pcVPC	prediction-corrected visual predictive check
PD	pharmacodynamic
P-gp	P-glycoprotein
PK	pharmacokinetics
PKPD	pharmacokinetic-pharmacodynamic
PM	poor metaboliser
PopPK	population pharmacokinetic
PP1M	paliperidone palmitate monthly
PP3M	paliperidone palmitate quarterly
PP6M	paliperidone palmitate half.yearly
PRISMA	Preferred Reporting Items for Systematic Reviews and Meta-Analyses
Q	apparent intercompartmental flow
RI	renal impairment
RIA	radio immunoassay
RISP	risperidone
RS	rich sampling
SAEM	stochastic approximation expectation-maximisation
SC	subcutaneous
SD	standard deviation
SI	strong CYP2D6 inhibitor
SS	sparse sampling
tlag	time delay on absorption
UM	ultra rapid metabolisers
V	apartment volume of distribution
Vd	volume of distribution
VPC	visual predictive check
WT	weight
